# Sirtuins and their role in ovarian aging-related fibrosis predisposing to ovarian cancer

**DOI:** 10.1038/s41514-025-00256-7

**Published:** 2025-07-15

**Authors:** Arkadiusz Grzeczka, Agnieszka Skowronska, Sara Sepe, Mariusz T. Skowronski, Paweł Kordowitzki

**Affiliations:** 1 Department of Basic and Preclinical Sciences, Faculty of Biological and Veterinary Sciences, Nicolaus Copernicus University, Torun, Poland; 2 Department of Human Physiology and Pathophysiology, School of Medicine, University of Warmia and Mazury in Olsztyn, Olsztyn, Poland; 3 IFOM ETS - The AIRC Institute of Molecular Oncology, Milan, Italy; 4 Department of Gynecology Including Center of Oncological Surgery (CVK), Charité Medical University, Berlin, Germany

**Keywords:** Cell biology, Biomarkers, Pathogenesis, Risk factors

## Abstract

The pursuit of understanding early genetic or protein markers for ovarian aging has garnered considerable attention in the realm of reproductive medicine. Sirtuins (SIRTs) are a group of proteins that are NAD^+^-dependent, and thanks to their properties, they are able to change the acetylation profile of proteins and post-translationally modify their functions, too. Previous research provided evidence that SIRTs influence fibrosis levels in several organs. With regard to ovaries, fibrosis is one of the features of aged ovaries and also creates a metastasis-friendly environment, thus can also be a seedbed for the development of primary cancerous lesions. Ovarian cancer remains a formidable challenge in oncology due to its high prevalence, insidious onset, and frequent recurrence. Noteworthy, ovarian cancer is the seventh most common cancer among women and the eighth leading cause of cancer death worldwide. Ovarian fibrosis runs concurrently with the activation of TGF-β/Smads signaling, as well as inflammasome (NLRP3), nuclear factor kB (NFkB) and forkhead box O (FOXO) attenuation. Reduced levels of certain sirtuins resulting from decreased nicotinamide adenine dinucleotide (NAD + ) may underlie the dysregulation of the aforementioned signaling pathways and therefore represent a potential therapeutic target. This review elucidates the role of SIRTs in ovarian aging-related fibrosis as a process that predisposes to tumorigenesis.

## Introduction

**Fig. 1:**
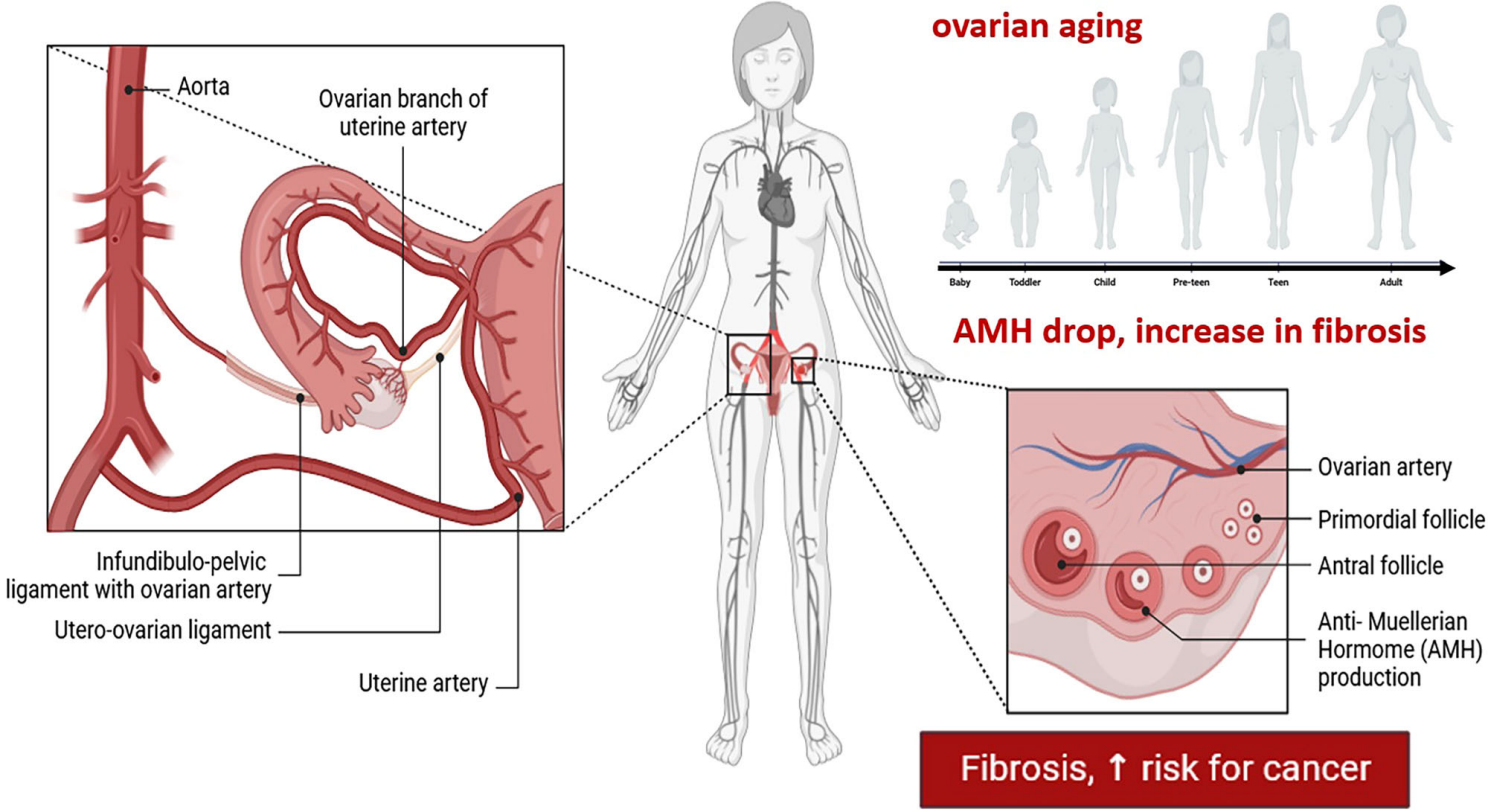
Scheme showing the phenomenon of ovarian aging and its effects on the decrease of the antral follicle count (AFC), and of the anti-Muellerian Hormone (AMH) in the bloodstream of women with advancing age. With increasing age, symptoms of fibrosis increase, and by this, the risk for tumorigenesis is elevated, too.

In the quest to comprehend the complexities of the aging phenomenon in the female reproductive system, early markers for ovarian aging, fibrosis, and cancer offer critical insights into the intricate cellular pathways that deteriorate as women’s ovaries age, presenting substantial implications for fertility treatments and reproductive health strategies (Fig. 1). Among various regulators of cellular homeostasis, the sirtuin (SIRT) family—comprising seven NAD⁺-dependent enzymes, SIRT1–SIRT7—acts as crucial sensors of energy and redox status^1^.

SIRTs are a family of seven enzymes: SIRT1-7, that play a key role in regulating protein function through post-translational modifications, primarily by removing acetyl groups^2^. Their roles in controlling oxidative stress, genomic stability, and cell fate position them as central modulators of both ovarian fibrosis and tumorigenesis. SIRT1 is widely recognized for its antifibrotic and tumor-suppressive effects through regulation of TGF-β, p53, and NF-κB pathways, although in certain cancers, its overexpression may support tumor progression^3–6^. SIRT2 regulates cell division and spindle integrity and has been shown to impact fibrotic signalling via DKK1 and Smad3, while also displaying tumor-suppressive properties in ovarian cancer^7–9^. SIRT3, the principal mitochondrial deacetylase, protects against oxidative stress and supports mitochondrial function; its decline with age promotes fibrosis and is associated with poor ovarian cancer prognosis^10,11^. SIRT4, though less well characterized, influences oocyte maturation and may contribute to mitotic stability; its aberrant expression is linked to both ovarian aging and malignancy^12–14^. SIRT5, through its desuccinylase activity, modulates metabolic enzymes involved in redox balance; it may promote chemoresistance in ovarian tumors and has been found altered in fibrotic ovarian conditions^15–17^. Nuclear sirtuins SIRT6 and SIRT7 are vital for chromatin remodeling and DNA repair; SIRT6 has been shown to counteract fibrotic matrix deposition and oxidative stress, while SIRT7 supports oocyte quality and genome maintenance, both showing deregulation in ovarian aging and cancer^18–22^.

As NAD⁺ levels decline with age, the activity of all SIRTs diminishes, contributing to dysregulation of cellular stress responses, extracellular matrix remodeling, and ultimately creating a microenvironment that favors both fibrosis and tumor development^23–26^. Age-related ovarian dysfunction has been linked to decreased levels of SIRTs^27^, i.e., decreased ovarian reserve, which has been associated with decreased levels of SIRT1, SIRT3 and SIRT6^26^. Another feature of aging is ovarian fibrosis^28^, and, as mentioned earlier, fibrosis can promote conditions similar to the pre-metastatic niche^29^. Tumor-associated fibrosis may have a variety of functions, including a role in drug resistance. Indeed, targeting Transforming growth factor-β (TGF-β), which leads to inhibition of fibrosis, can sensitize the tumor to immunotherapies^30,31^. However, some studies claim that ovarian fibrosis may contribute to the development of a microenvironment favourable for tumor growth by mobilizing ECM components, primarily collagen, and activating fibrotic pathways, e.g. TGF-β, which are closely related to the potential of cells to undergo the EMT process. Interestingly, most malignant tumors are accompanied by severe stromal changes^32^, and increasing stiffness of ovarian tissue facilitates tumor invasion or metastasis^33^. Fibrotic ovaries are also encountered in postmenopausal and advanced-age women, a period of life that predisposes to the development of ovarian cancer (OC)^34^. The age-related accumulation of damage caused by increasing oxido-inflammatory stress contributes to worsening changes in the ovarian stroma and capsule, which may be linked to a higher predisposition to OC^29^.

This review aims to explore the multifaceted roles of sirtuins in ovarian aging-related fibrosis and their contribution to ovarian cancer predisposition, integrating their molecular actions within key signaling pathways and physiological contexts.

## Sirtuins and TGF-β and ECM modifications in ovarian fibrosis

**Fig. 2:**
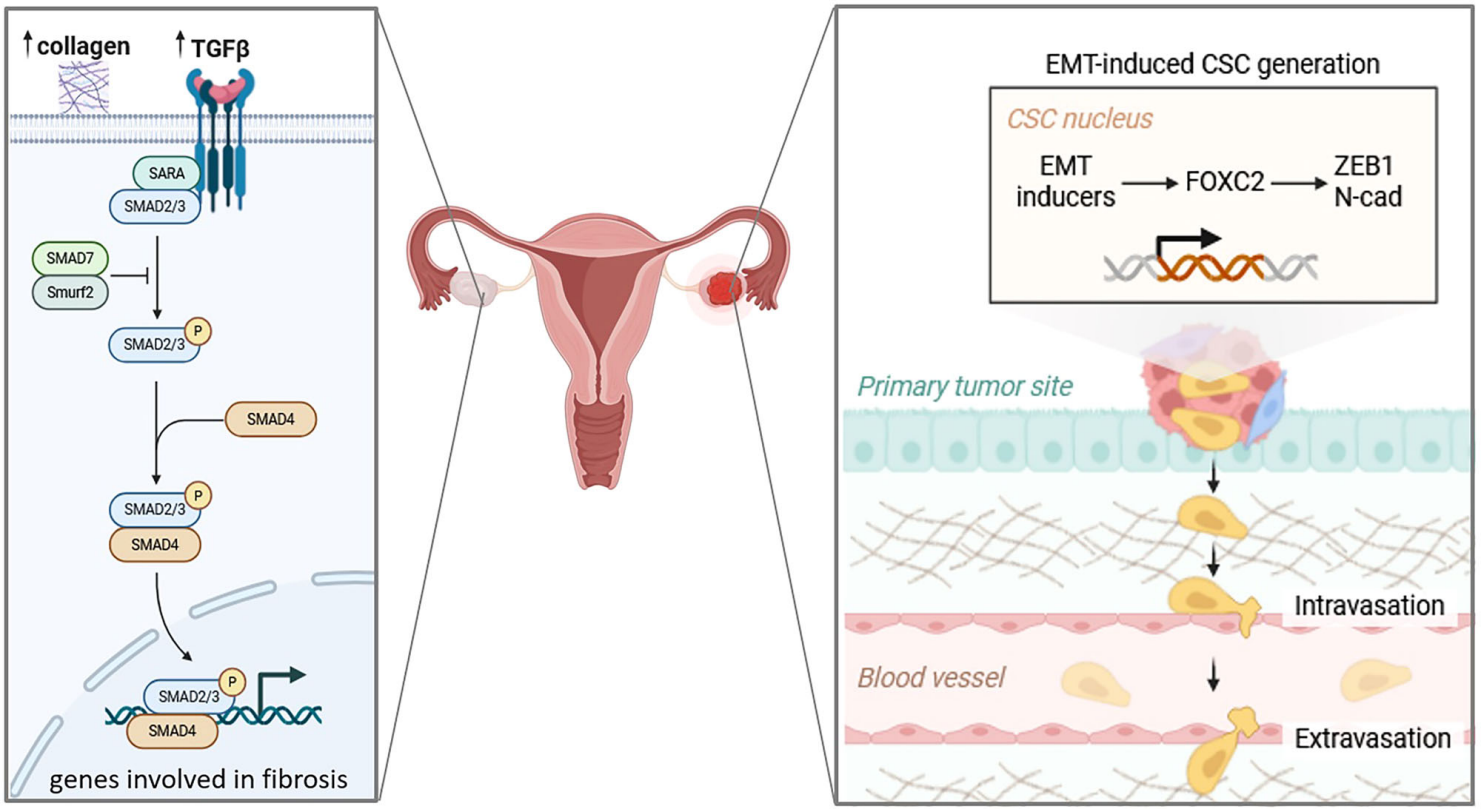
Scheme showing the epithelial-to-mesenchymal transition (EMT) and the intravasation of cancer stem cells (CSC) from the primary tumor site into the bloodstream and their extravasation. Metformin therapy lowers TGFβ2 expression, and in ovaries of postmenopausal women have higher TGFβ and collagen expression. Metformin causes the downregulation of genes involved in fibrosis and the creation of the premetastatic niche.

Transforming growth factor-β (TGF-β) is a major signaling pathway responsible for the activation of fibroblasts, involved in fibrosis in many organs, including the heart, lung, liver, and ovaries^35^. In recent years, research has significantly focused on ovarian fibrosis as a feature of aging^36,37^ and the associated predisposition to tumorigenesis^38^. TGF-β also controls the pathways responsible for epithelial-to-mesenchymal transition (EMT) (Fig. 2), endothelial-to-mesenchymal transition (EndMT), and Wnt/β-Catenin - key fibrosis pathways during carcinogenesis^39,40^. Given that the acetylation level of Small Mother Against Decapentaplegic (Smads) transcription factors is crucial for the events\triggered by TGF-β receptor 2 (TGFR2) and TGFR1 activation, sirtuins, as constitutive deacetylases, may play a key role in regulating the activation of Smad2, Smad3, Smad4 and Smad7 and translocation of the Smad2/3 complex to the nucleus^41^. Interestingly, the dynamics of sirtuin activity may differ from one ovarian cell to another, as with age, the SIRTs activity is attenuated in granulosa cells and increased in oocytes, suggesting a different role for each cell in aging processes and a possible influence on fibrosis progression^42^ [Table 1]. Smads deficiency or dysfunction can have consequences on granulosa cell function as well as on thecal cells, causing fertility defects^43^. Interestingly, the thecal cells, which are the first ovarian cells to up-regulate fibrosis triggered by the TGFB1, TGFB2, and Smad3 as a result of aging, may play a key role in fibroblast activation^42^. TGF-β target genes involved in ovarian fibrosis include alpha-smooth muscle actin (α-SMA) and Col1a1, Col1a2, and Col3a1. While the first is a marker of myofibroblasts and activated fibroblasts, the latter are the main genes responsible for remodelling the ECM into excessive collagen and thickening its fibres. The expression of those genes increases with age, and they have a role also in the pathogenesis of other ovarian diseases, such as PCOS. Several populations of fibroblasts at different levels of differentiation and myofibroblasts with high α-SMA expression have been demonstrated in aging mice^44^. Even though a differentiated pool of fibroblasts represents a physiological condition of a dynamic cycle enabling dependent on TGF-β signaling, the old mice showed an increased proportion of fibroblasts with a senescence-associated secretory phenotype (SASP)^44^. Increased ovarian collagen deposition correlates with a decrease in SIRT1 expression in ovaries with PCOS^45,46^, with a decrease in nicotinamide adenine dinucleotide (NAD + ) levels with age in ovaries^47,48^, and with a decrease in SIRT1 expression and reduced ovarian reserve^49^. One of the few studies investigating the direct relationship of SIRT1 with Smads in ovaries shows that resveratrol downregulates Smad2/3 phosphorylation and inhibits follicle-stimulating hormone (FSHβ) expression, but inhibition of SIRT1 did not affect the efficacy of resveratrol^50^, suggesting that SIRT1 does not directly control the phosphorylation of Smad2/3. However, one limitation of this observation is the experimental plan that involves immortalized cells which may have influenced the results, but it is possible that SIRT1 or resveratrol itself only indirectly affects TGF-β pathways by reducing oxidative stress, process clarified in the following paragraph^50,51^. On the other hand, it has been found that in granulosa cells, TGF-β/Smad3 regulation is mediated by SIRT2 via Dickkopf deacetylation 1 (DKK1)^9^. DKK1 is a mediator of both cancerous and non-cancerous diseases, including fibrosis-depend diseases such as ischaemic heart disease^52^. In polycystic ovary syndrome (PCOS), overexpression of DKK1 reduced the apoptosis rate of granulosa cells; moreover, overactivation of SIRT2 and increased deacetylation of DKK1 were detected in cells from PCOS rats. In confirmation of this, the SIRT2 inhibitor, AGK2, induced similar effects to DKK1 overexpression^9^.

**Table 1:** The role of sirtuin in the ovary and its relationship with age

	Role in the ovary	Age-related changes	Key molecular pathways/functions
**SIRT1**	Regulates oocyte maturation, oxidative stress, apoptosis, and maintenance of granulosa cell quality^149^.	.Decreased expression with age; lower levels correlate with deterioration in oocyte quality and mitochondrial function^91,150^	FOXO, p53, NF-κB, oxidative stress regulation, apoptosis pathways^151^.
**SIRT2**	.Participates in the control of cell division, spindle stability and deacetylation of α-tubulin^152,153^	.Reduced expression in aging oocytes; associated with increased risk of aneuploidy^154,155^	Cdk1/cyclin B, α-tubulin, cell cycle regulation, spindle organization^9^.
**SIRT3**	Regulates mitochondrial metabolism and protection against oxidative stress in oocytes^10^.	Declines with age; its loss impairs mitochondrial quality and increases oxidative damage^10^.	OXPHOS pathways, MnSOD deacetylation, ROS regulation^10^.
**SIRT4**	Participates in energy metabolism in mitochondria^13^.	No clear data on age-related changes^156^, but low expression reported in ovarian cancer^14^. In contrast, higher levels were detected in older mice, and their knockdown restored normal spindle organisation^13^.	Modulation of PDHE1α subunit phosphorylation, Chromosome segregation^13^.
**SIRT5**	Regulates metabolic pathways through desuccinylation and demalonylation of mitochondrial proteins^157^.	May be overexpressed in early ovarian tumorigenesis^16^, or reduce in women with reduced ovarian reserve^17^.	TCA cycle, ammonia detoxification, cisplatin resistance^16^.
**SIRT6**	Regulates oocyte maturation, cumulus cell expansion, and spindle organization^20^.	Increased expression of SIRT6 in ovarian cancer indicates a better prognosis^14^.	DNA repair, gene expression via H3K9Ac and H3K56Ac, CDK1, cumulus expansion pathways^20^.
**SIRT7**	Maintains genome stability, promotes chromosome synapsis and DNA repair in oocytes^19^.	Decreases with age; SIRT7 knockout leads to lower oocyte quality and higher aneuploidy^19^.	DNA repair (γH2AX, MLH1), H3K18Ac regulation, crossover control^158^.

Sirt6 levels, which also decline with age, guarantee adequate ovarian stroma development^22^. Indeed, SIRT6 knock-out mice show decreased expression of Plod1, the gene responsible for encoding the main collagen synthesizing enzyme, lysyl hydroxylase. Downregulation of lysyl hydroxylase led to ovarian hypoplasia and reduced collagen fibre thickness^22^. This evidence corroborates the role of SIRTs in ovarian fibrosis. Resveratrol treatment attenuates fibrinogen signaling and interstitial fibrosis by decreasing TGF-β, α-SMA, and type IV collagen deposition, thereby contributing to increased luteal cells and antral follicles^51^. Targets of the TGF-β/Smad2/Smad3 axis (Fig. 2), responsible for proliferation, migration, and differentiation, are also activated by induction of β-catenin nuclear translocation by TGF-β^53^. Stimulation of SIRT1 expression reduces β-catenin levels in androgen-induced fibrosis^51^.

Additionally, positive effects of metformin have been indicated in mice with ovarian fibrosis^44^, ovarian fibrosis in postmenopausal women^54^, and ovaries affected by PCOS^55^. Metformin is an agonist with direct effects on SIRT1^56^ and also AMP-activated protein kinase (AMPK)^57^. The ovaries of postmenopausal women have higher TGF-β and collagen expression (Fig. 2), and the metformin administration reduces TGF-β2 expression^54^. In addition, metformin leads to the down-regulation of genes associated with fibrosis and pre-metastatic niche formation^54^. A study in aged mice showed that metformin was effective in preventing fibrosis and less effective in curing fibrosis that occurred prior to supplementation^44^. A study comparing the effects of metformin and resveratrol in mice with PCOS shows that both treatments do not affect the expression of SIRT1 and AMPK (target genes for resveratrol and metformin, respectively), yet equally improved ovarian structure with a consequent increase in corpus luteum and graft follicles^58^. Those results indicate a key role for AMPK/SIRT1 signalling in counteracting ovarian fibrosis. There is a known relationship between AMPK and SIRT1, in which deacetylase is required for the activation of liver kinase B1 (LKB1), which phosphorylates the α-AMPK subunit. In the case of metformin, however, it has been shown that its anti-fibrotic effect is not related to an effect on the metabolism of abnormal ovarian fibroblast populations (highly active α-SMA-labelled) but rather modulates immune status^44^ as it is described below. The greatest limit to the study of the association of SIRTs with major fibrotic pathways, such as TGF-β, is still the scarce knowledge of the distribution and the age-related changes of TGF-β levels in the different ovarian cells, which can be very different based on the cell type^59^.

## Sirtuins and inflammation in ovarian fibrosis

Ovarian fibrosis is increasingly recognized as a hallmark of ovarian aging and pathology, including polycystic ovary syndrome (PCOS) and ovarian cancer. Fibrosis-related pathways are activated with age, particularly those associated with chronic inflammation. In aged ovarian tissue, there is notable upregulation of inflammatory mediators such as TNF-α, IL-1β, IL-6, and components of the NLRP3 inflammasome^37,44,60^. These cytokines are secreted not only by resident ovarian cells but also by infiltrating immune cells and senescent fibroblasts that exhibit a senescence-associated secretory phenotype (SASP)^44^. The NF-κB signaling pathway plays a central role in sustaining this inflammatory environment. It promotes the expression of proinflammatory genes and contributes to fibrotic remodelling by enhancing fibroblast activation. Importantly, fibroblasts isolated from aged ovaries show enrichment in NF-κB pathway activity compared to those from young, non-fibrotic tissue^44^. Similarly, the TGF-β pathway, which intersects with both NF-κB and JAK/STAT signaling, contributes to fibroblast-to-myofibroblast transition and collagen deposition. STAT3, in particular, is activated by cytokines such as IL-6 and plays a critical role in both systemic fibrotic diseases and ovarian cancer^61,62^. In addition to stromal alterations, the ovarian immune microenvironment undergoes age-related remodeling. Immunosenescence, characterized by a shift from CD4+ to CD8 + T cells and increased M2 macrophage polarization, contributes to a pro-fibrotic state^63,64^. Chemokines such as CCL2 and CCL5, involved in leukocyte recruitment, are also upregulated with age^37^. Multinucleated macrophages, another feature of aged ovaries, may serve as biomarkers of fibrotic progression^65^. Sirtuins, particularly SIRT1, SIRT2, SIRT3, and SIRT7, modulate many of these inflammatory and fibrotic pathways. SIRT1 negatively regulates NF-κB by deacetylating its p65 subunit, thereby reducing its transcriptional activity and dampening inflammation^66^. Treatments that activate the AMPK/SIRT1 axis, such as myo-inositol and semaglutide, have been shown to reduce inflammatory cytokines and fibrosis in PCOS models and aged ovaries^45,67^. Likewise, resveratrol, a potent SIRT1 activator, decreases NF-κB/p65 activity and alleviates ovarian inflammation^58,68^. SIRT2 expression increases following nicotinamide mononucleotide (NMN) supplementation and is associated with reduced levels of NLRP3, IL-18, and IL-1β in aged ovaries, indicating its anti-inflammatory potential^69^. Furthermore, SIRT3 negatively correlates with STAT3 activity in ovarian cancer, suggesting a suppressive role in inflammation-driven tumorigenesis^62^. Lastly, SIRT7 has been shown to interact with both p65 and p50 subunits of NF-κB, modulating its expression and activity in ovarian cancer cells^21^. Together, these findings highlight the interconnected roles of inflammatory pathways and sirtuins in the progression of ovarian fibrosis and inflammation. Understanding these interactions opens new avenues for therapeutic interventions targeting aging-related ovarian disorders.

These findings underscore the multifactorial nature of ovarian fibrosis, where chronic inflammation, immune system remodeling, and stromal cell activation converge to drive progressive fibrotic changes in aging ovarian tissue. Sirtuins emerge as key molecular regulators within this network, exerting anti-inflammatory and antifibrotic effects through modulation of NF-κB, STAT3, and inflammasome signaling. Their interaction with metabolic pathways such as AMPK and their responsiveness to NAD+ availability suggest a broader role in linking energy status with immune and fibrotic responses. Continued investigation into the specific mechanisms by which distinct sirtuin isoforms influence these processes may yield novel insights into ovarian aging and identify potential molecular targets for therapeutic modulation in fibrotic and inflammation-associated ovarian disorders.

## Sirtuins and oxidative stress in ovarian fibrosis

Ovarian fibrosis is a hallmark of reproductive aging and a common feature in pathological conditions such as premature ovarian insufficiency and polycystic ovary syndrome. One of the central mechanisms contributing to this fibrotic remodelling is oxidative stress (OS), which arises from an imbalance between the production of reactive oxygen species (ROS) and the cell’s ability to detoxify them. ROS can damage lipids, proteins, and nucleic acids, and their accumulation in ovarian cells impairs mitochondrial function, disrupts hormonal signaling, and promotes the activation of fibrotic and inflammatory pathways.

A primary target of oxidative damage is telomeric DNA, which, due to its guanine-rich sequence and limited repair capacity, is especially susceptible to ROS-induced breaks. This damage activates the DNA damage response (DDR) via pathways involving γH2AX, XRCC6, and PARP1, which are upregulated in aging ovaries^70–73^. Telomere shortening and dysfunction consequently promote cellular senescence and apoptosis^74,75^, processes that drive tissue remodeling and fibrotic changes. Additionally, OS and DNA damage induces activation of p53, which transcriptionally regulates pro-apoptotic and pro-fibrotic genes, often through upregulation of CDKN1A (p21) and downregulation of the retinoblastoma protein (RB) pathway, leading to cell cycle arrest and promoting senescence-associated secretory phenotypes (SASP)^76^. Another major contributor to fibrotic remodeling under OS is p66Shc, a redox enzyme that promotes mitochondrial ROS production and upregulates profibrotic markers such as α-SMA and NLRP3 inflammasome components^77^. p66Shc levels increase with age and correlate with fibrosis in the ovary and other organs. Deletion of p66 in progeric mice (telomerase RNA component knockout mice) alleviates age-related phenotypes, indicating a potential role for p66 in the ageing process^78^. Similarly, dysregulation of the Keap1/Nrf2 antioxidant pathway under prolonged OS limits the cellular ability to activate antioxidant genes such as HO-1, SOD2, and CAT. Normally, oxidative stress leads to dissociation of Nrf2 from Keap1, its nuclear translocation, and activation of antioxidant response elements (ARE), but this axis is impaired in several ovarian pathologies including PCOS^79–81^. Mitochondrial dysfunction, another key element in fibrogenesis, is tightly coupled to cellular redox status. With aging, mitochondrial membrane potential and oxidative phosphorylation efficiency decline, NAD + /NADH ratios drop, and ATP production is reduced. This leads to an increase in mitochondrial ROS and subsequent oxidative damage, further driving fibrotic gene expression and cellular dysfunction^82,83^.

In this context of pro-fibrotic and oxidative signaling, sirtuins act as crucial regulators that can suppress or reverse many of these pathological changes. These NAD + -dependent deacetylases respond to metabolic and redox status and coordinate protective mechanisms against OS and fibrosis. Among them, SIRT1, SIRT3, SIRT5, SIRT6, and SIRT7 have been most widely studied in ovarian tissue. SIRT1 regulates the DDR by deacetylating p53, thereby limiting its pro-apoptotic and pro-fibrotic activity^5,6^. Under oxidative conditions, SIRT1 expression is upregulated as a compensatory response, and its activity is further enhanced by compounds like celastrol and melatonin, which reduce the levels and activaion of γH2AX, XRCC6, and PARP1^84,85^. Moreover, the kinase TOPK promotes SIRT1 expression while repressing p53 acetylation, and its inhibition leads to apoptosis under inflammatory stress, indicating the importance of this regulatory axis in follicular survival^86,87^. SIRT1 also suppresses p66Shc expression, thereby attenuating ROS generation and inflammasome activation. In models of hyperandrogenism-induced ovarian fibrosis, resveratrol treatment upregulates SIRT1, reduces p66Shc levels, and ameliorates fibrotic changes^51^. SIRT6 complements this function by repressing p66Shc promoter activity and deacetylating histone H3K9Ac, reducing transcription of pro-apoptotic and pro-fibrotic genes^88–90^. SIRT3 and SIRT5, both localized in mitochondria, maintain mitochondrial integrity and redox balance. SIRT3 deacetylates and activates FOXO3a, which in turn upregulates antioxidant enzymes such as SOD2 and CAT^91–94^. It also regulates PGC-1α and TFAM, promoting mitochondrial biogenesis. In PCOS and aging models, SIRT3 expression is reduced, correlating with decreased antioxidant defense and increased fibrotic gene expression^95–97^. Resveratrol and melatonin restore SIRT3 levels and improve mitochondrial function, reversing these pathological changes^98,99^. SIRT5, through its desuccinylase activity, regulates mitochondrial enzymes involved in oxidative phosphorylation and supports redox homeostasis, although its specific role in ovarian fibrosis remains to be fully elucidated^100^. SIRT7, although less extensively studied in the context of ovarian function, has emerged as a key regulator of mitochondrial homeostasis and resistance to oxidative stress. It promotes mitochondrial ribosomal protein expression, supports mitochondrial translation, and limits the accumulation of ROS^101^. Importantly, the miR-17-5p/SIRT7 axis is a key regulatory factor in the DNA damage response in the ovaries, and its influence has been shown to reduce OS and the levels of γH2AX, XRCC6, and PARP1 activity^84^. Reduced expression of SIRT7 with aging may thus contribute to mitochondrial decline and the establishment of a pro-fibrotic environment. The interaction between sirtuins and FOXO transcription factors is central to the cellular oxidative stress response. SIRT1 and SIRT3 promote FOXO1 and FOXO3a activity, enhancing resistance to oxidative damage and inhibiting apoptosis^91–94^. Disruption of this interaction by miRNAs such as miR-132 and miR-181a leads to FOXO inactivation, increased apoptosis, and promotion of fibrotic and neoplastic changes^91,102,103^. Sirtuins also influence the Nrf2 pathway. SIRT1 deacetylates Nrf2, facilitating its nuclear translocation and activation of antioxidant genes. Natural compounds such as icariin and resveratrol amplify this effect, enhancing the expression of protective enzymes like HO-1 and restoring redox balance in oxidative ovarian environments^104–106^. Finally, declining NAD+ levels during aging limit sirtuin activity and thereby compromise antioxidant defenses and mitochondrial function. Supplementation with NAD+ precursors like nicotinamide riboside improves ovarian function by restoring NAD+ pools, increasing SIRT1 and SIRT3 expression, and improving mitochondrial energy metabolism^47,48,95,107–109^.

Sirtuin-mediated pathways therefore intersect with multiple regulatory networks involved in oxidative stress, mitochondrial function, and the fibrotic remodeling of ovarian tissue. Their activity is tightly linked to cellular energy status and redox balance, positioning them as dynamic sensors and modulators of homeostasis in the ovarian microenvironment.

## Ovarian cancer and sirtuins

High-grade serous ovarian cancer (HGSOC) represents one of the most challenging and lethal forms of gynecological malignancies, characterized by its aggressive progression and often late-stage diagnosis.

Genetic predispositions play a crucial role in the etiology of HGSOC. As recently shown, the prevalence of HGSOC subtype-specific survival varies by race^110^. Mutations in the BRCA1 and BRCA2 genes are the most significant genetic factors associated with an increased risk of developing ovarian cancer. Women carrying BRCA1 mutations face a 39%-44% lifetime risk, while BRCA2 mutation carriers have an 11%-17% lifetime risk of developing ovarian cancer (American Cancer Society). Besides BRCA mutations, other genetic factors such as mutations in BRIP1, RAD51C, and the genes associated with Lynch syndrome (PMS2, MLH1, MSH2, and MSH6) also contribute to the risk profile for HGSOC^111–113^. Familial aggregation of ovarian and other cancers, such as breast, pancreatic, melanoma, and colon cancers, in first-degree relatives, underscores the importance of genetic epidemiology in HGSOC. Understanding these genetic predispositions is essential for identifying high-risk individuals and implementing preventive measures such as genetic counselling and risk-reducing surgeries.

**Fig. 3:**
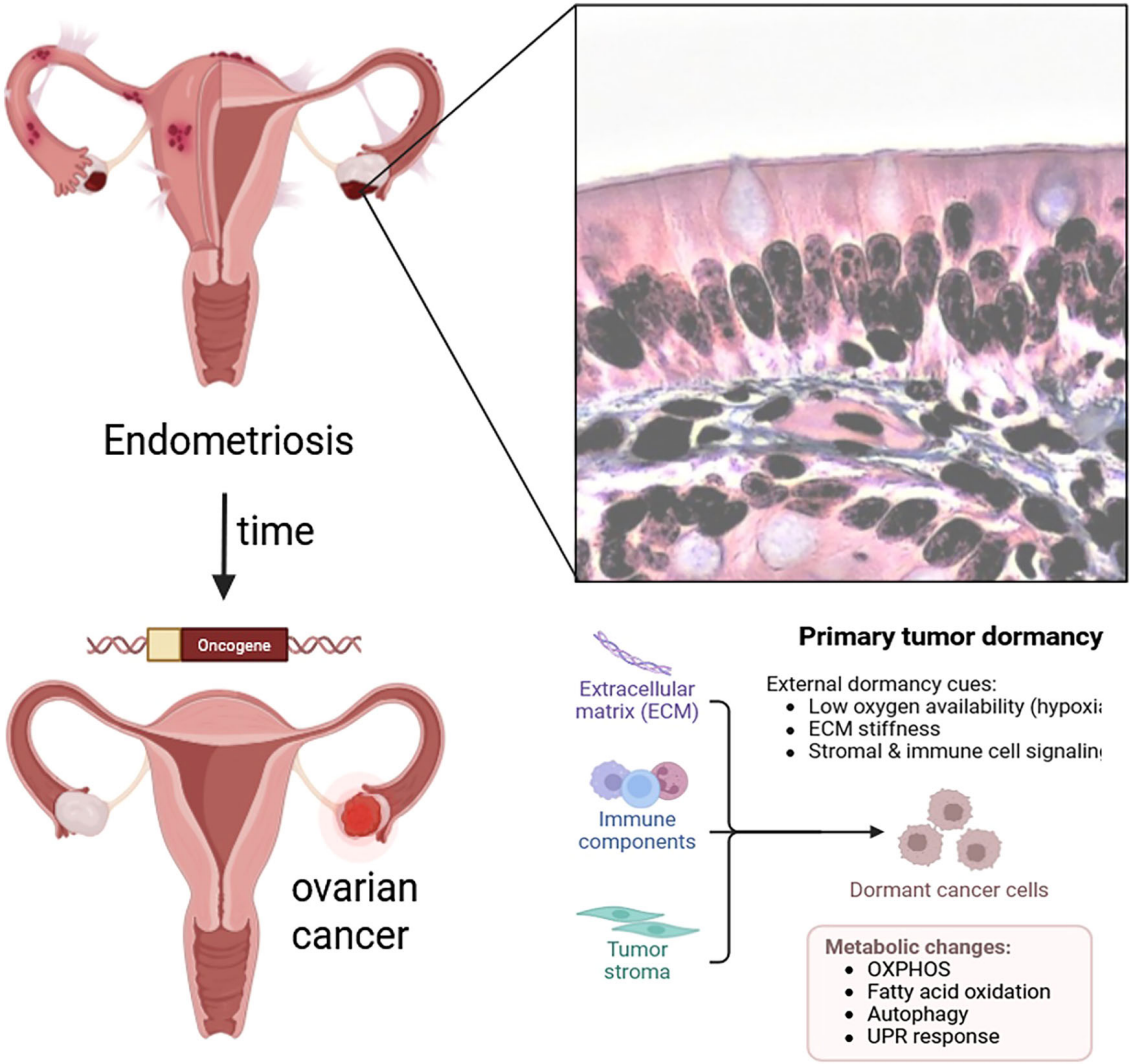
Scheme showing the development of ovarian endometriosis into ovarian cancer and the primary tumor dormancy.

Environmental and lifestyle factors are also implicated in the epidemiology of HGSOC. Obesity, use of hormone replacement therapy (HRT), and reproductive history, including lower parity, lifetime number of ovulatory cycles, and infertility, have been identified as significant risk factors for developing ovarian cancer^112,114^. While combined oral contraceptive use lowers the risk of HGSOC, the use of postmenopausal hormone therapy slightly increases the risk for HGSOC, highlighting the complex interplay between hormonal influences and cancer development^112,115^. Dietary factors, and physical inactivity are additional lifestyle factors that may contribute to ovarian cancer risk. For instance, diets high in fats and low in fruits and vegetables are associated with higher cancer risk, while regular physical activity may offer protective benefits^111,112^. Environmental exposures such as the use of talcum powder in the genital area and prolonged exposure to asbestos have also been studied for their potential to increase ovarian cancer risk^111,112^. The incidence of epithelial ovarian cancer, particularly endometriosis-associated ovarian cancer such as clear cell carcinoma and endometrioid carcinoma, has markedly increased in Japan^116^. It has also been reported that clear cell carcinoma and endometrioid carcinoma frequently co-exist with endometriosis, suggesting that endometriosis is a possible precursor lesion for these types of ovarian cancer. Endometriosis, a chronic gynecological condition affecting approximately 176 million ( ~10%) women of reproductive age worldwide, is another common risk factor for some OC types^117^. In a recent study in which 450 906 patients with and without endometriosis were analyzed, a history of endometriosis conferred a 4.2-fold increased risk for ovarian cancer^118^. Noteworthy, patients suffering from ovarian endometriomas and/or deep infiltrating endometriosis showed a 9.7-fold higher risk when compared to counterparts without endometriosis (Fig. 3)^118^. However, the risk for endometriosis patients to develop high-grade serous ovarian cancers is lower (2.7%) than to develop other histotypes, such as, endometrioid (7.96%) or low-grade serous (8.12%) ovarian cancer^118^. Women suffering from endometriosis are concerned about the increased ovarian cancer risk, and endometriosis-associated ovarian cancer is challenging for clinicians^118^. Moreover, the aforementioned patients are suffering from pain, anxiety and depression, impacting their psychological and social functioning^119,120^. Counselling programs for patients suffering from endometriosis should also be provided to test for specific gene mutations that could later in life cause the development of ovarian cancer^117,118^. Recent bioinformatic analyses have revealed a significant molecular overlap between PCOS and ovarian cancer, suggesting that PCOS may serve as a precursor condition for some OC subtypes. A set of 128 differentially expressed genes was found to be common to both PCOS and OC, with particular emphasis on OGN (osteoglycin) as a potential biomarker linking the two^121^. Lower OGN expression, frequently observed in both PCOS and OC tissues, was associated with altered hormone signaling and poor prognosis, potentially promoting tumor progression through dysregulation of FSHR and m6A methylation. These findings imply that PCOS, particularly when marked by hormonal imbalance and genetic susceptibility, may contribute to the molecular pathogenesis of ovarian cancer^121^.

In the context of OC, the role of sirtuins remains an active area of investigation. SIRT1 functions as an oncogene as well as a tumor suppressor, regulating cell cycle progression, apoptosis, cell senescence, and oxidative stress resistance. Several studies have linked SIRT1 to cancer stemness and resistance to conventional therapy. SIRT1 has been shown to play a complex and often contradictory role in cancer development and progression^122^. One study revealed that SIRT1 expression was significantly decreased in OC tissues compared to normal ovarian tissues, and that lower SIRT1 levels were associated with more advanced disease stages and poorer patient survival^123–125^. In another, SIRT1 overexpression increases chemoresistance, tumorigenesis and epithelial-to-mesenchymal transition (EMT) phenotype^126,127^. By extension, overexpression may promote a poorer prognosis for patients with OC^11,123,128^. SIRT1 inhibition generally promotes the survival, proliferation, and metabolism of cancer cells, playing an important role in their resistance to treatment. However, MHY2245, a new SIRT1 inhibitor, by inhibiting the activity and expression of SIRT1, leads to cell cycle arrest, apoptosis, and autophagy in cancer cells^129^. Extracellular vesicles derived from cancer-associated adipocytes (CAA-EVs) play a crucial role in ovarian cancer progression by modulating the immune response and tumorigenesis^130^. These vesicles carry SIRT1, which transcriptionally activates CD24 expression, leading to suppression of CD8 + T cell activity and promoting tumor immune escape^131^. Other studies indicate that SIRT1 plays a key role in inhibiting the progression of ovarian cancer by regulating the expression and acetylation of HMGB1. Importantly, overexpression of SIRT1 effectively reduced the migration and invasion of cancer cells and decreased angiogenesis, suggesting its therapeutic potential in the treatment of this aggressive disease^132^. Despite the original reports of an adverse effect of SIRT2 on OC prognosis^133^, several recent studies have indicated that it is an OC suppressor^11,123^. For example, lower expression of SIRT2 was associated with higher expression of cyclin-dependent kinase 4 (cdk4)^134^, and SIRT2 overexpression had a favorable effect on the prognosis of OC patients^123^. Metastatic spread is the main cause of death in epithelial ovarian cancer, yet the mechanisms remain unclear. Fn14 acts as a metastasis suppressor by inhibiting migration and invasion of EOC cells through downregulation of EMT^135^. Mechanistically, Fn14 promotes acetylation-dependent degradation of Slug, a key EMT transcription factor, by interfering with SIRT2. Fn14 binds SIRT2, preventing its nuclear entry and thus reducing Slug deacetylation and stabilization^135^. The third significantly down-regulated sirtuin in OC is SIRT3^11^. Since tumorigenesis destabilizes the cell’s energy economy, the role of SIRT3 may be crucial in OC. In one recent study, SIRT3 transcript levels in various OC subtypes were significantly lower than in normal tissues^123^. Moreover, as one of the most important proteins of mitochondrial metabolism, SIRT3, was identified as an independent favourable prognostic factor of OC^136^. Interestingly, downregulated SIRT3 has also been detected in pre-metastatic tissue^137^. SIRT3 is downregulated in metastatic ovarian cancer tissues and cells. Its knockdown enhances migration, invasion, and metastasis, while overexpression suppresses these processes. The mechanism involves inhibiting EMT by reducing the level of the protein Twist, with which SIRT3 directly interacts. The SIRT3/Twist axis may represent a novel therapeutic target for metastatic ovarian cancer^138^. Increased expression of mitochondrial proteins may occur in response to oxidative damage to cells in early tumorigenesis. SIRT5, which mainly acts in its territory, is also overexpressed in early tumorigenesis^139^. In addition, SIRT5 increases OC resistance to cisplatin^16^. Mechanistically, it suppresses cisplatin-induced DNA damage by reducing reactive oxygen species through activation of the Nrf2/HO-1 antioxidant pathway, thereby contributing to chemoresistance in ovarian cancer^16^. In some types of liver cancer, SIRT5 depression limits disease progression^140^. In ovarian cancer, reduction of SIRT5 expression, through upregulation of miR-27b-5p, reduced tumor progression in vitro^141^. The opposite dynamic is characterized by SIRT4, which maintains low escapes in OC^123,142^, but SIRT4 overexpression adversely affects OC prognosis^11^. The other sirtuins, nuclear SIRT6 and SIRT7, are lower in OC^123^. This may indicate that their function is impaired and that genome stability and DNA repair processes are impaired, which is characteristic of tumorigenesis progression^123^. Unfortunately, the results of other studies are inconclusive, on the one hand pointing to a suppressor character^143,144^ and on the other, promoting OC progression^145^. Sirtuins, especially SIRT4 and SIRT6, play opposing roles in regulating ovarian cancer cell survival, making them potential competitive prognostic biomarkers. Bioinformatic analyses and immunohistochemical studies have shown that their high expression levels are associated with different prognoses and distinct impacts on tumor progression. SIRT4 is involved in the immune response during oocyte maturation, while SIRT6 participates in regulating mitochondrial processes and immune-related diseases, indicating their involvement in conflicting mechanisms influencing disease development^14^. In p53-mutant ovarian cancer, tumor cells under cisplatin treatment release exosomes containing the long non-coding RNA PANDAR (correlates with poor prognosis and promotes the development of cancer), which binds to the protein SRSF9^146^. After translocation to the nucleus, SRSF9 suppresses apoptosis and modifies gene expression, leading to an altered mRNA ratio of SIRT4/SIRT6 that promotes cell survival and the development of cisplatin resistance. This mechanism enables tumor cells to rapidly adapt to treatment-induced stress, hindering therapeutic efficacy^147^. Understanding this complex interaction between PANDAR, SRSF9, and sirtuins could open new therapeutic avenues for treating cisplatin-resistant ovarian cancers.

## Conclusions

Sirtuins, through their extensive subcellular localization, exert important effects on energy (NAD + ) metabolism, on which cellular functions depend, and influence key signaling pathways directly related to ovarian fibrosis (Smads, TGF-β). As we have presented, the kinetics of SIRT1 and SIRT3 are the best understood so far. However, this is not surprising because of their indisputable effects on Smads pathway gene transcription (in the case of SIRT1) and mitochondrial metabolism (in the case of SIRT3). In the case of the remaining members, we can expect similar functions to SIRT1 from SIRT6 and SIRT7, due to their similar localization. The same situation applies to SIRT3 and SIRT5. While the functions of SIRT2 and SIRT4 in the ovary remain underexplored, future studies could investigate their potential roles by drawing parallels with their known activities in other tissues, such as SIRT2’s involvement in cell cycle regulation and microtubule dynamics, and SIRT4’s role in mitochondrial metabolism and stress response.

What has been established is that inhibition of the major inflammatory and oxidative stress pathways NLRP3, NFkB reduced the expression of fibrosis markers (α-SMA) in ovarian tissue from aging mice, mice with induced fibrosis as well as ovarian tissue from postmenopausal women. In addition, sirtuins collaborate with key pathways that maintain mitochondrial fitness and biogenesis (PGC-1α), and transcription factors (Nrf2, FOXO) that ensure adequate expression of antioxidant enzymes. As a separate observation, there is an interesting relationship between sirtuins and AMPK, which translates, for example, into modifications of the macrophage population, making it possible to discover the true causes of ovarian fibrosis. In the context of tumorigenesis, an interesting relationship has emerged between sirtuins and important fibrotic pathways for OC. Indeed, sirtuins can model the EMT process, a key process for metastasis and overexpression of sirtuins was able to reduce collagen deposition in ageing mice. While therapeutic agents like metformin (an AMPK and SIRT1 agonist) demonstrate potential in mitigating ovarian fibrosis, and resveratrol may offer even stronger SIRT1 activation, further research is needed to address limitations such as side effects and the scarcity of robust human clinical data supporting their efficacy and safety. The action of resveratrol is limited by its low bioavailability, and its interaction with other drugs (due to the inhibition of cytochromes P450) is still poorly understood^148^. Metformin, on the other hand, can lead to numerous disorders of the digestive and endocrine systems^148^.

## Data Availability

No datasets were generated or analysed during the current study.
